# Attitudes toward psychedelic therapy among medical and nursing students: A cross-sectional survey study

**DOI:** 10.1371/journal.pone.0344698

**Published:** 2026-03-31

**Authors:** Diego Castellano-Ramírez, Elisa Hernández-Álvarez, Lucas F. Borkel, Jaime Rojas-Hernández, Domingo Quintana-Hernández, Luis Alberto Henríquez-Hernández

**Affiliations:** 1 School of Nursing, Faculty of Health Sciences, Universidad de Las Palmas de Gran Canaria, Las Palmas de Gran Canaria, Spain; 2 Unit of Toxicology, Clinical Sciences Department, Universidad de Las Palmas de Gran Canaria, Las Palmas de Gran Canaria, Spain; 3 Asociación Científica Psicodélica, Arucas, Spain; 4 Universidad Fernando Pessoa Canarias, Guía, Spain; 5 Asociación Canaria para el Desarrollo de la Salud a Través de la Atención, Las Palmas de Gran Canaria, Spain; 6 Faculty of Psychology, Universidad del Atlántico Medio, Las Palmas de Gran Canaria, Spain; Federal University of Rio Grande do Norte: Universidade Federal do Rio Grande do Norte, BRAZIL

## Abstract

Psychedelic-assisted therapy is regaining attention as a promising approach to treating mental health disorders. This research aimed to compare related factors regarding the therapeutic use of psychedelics between medical and nursing students, addressing a notable gap in existing research. A cross-sectional survey was conducted at the University of Las Palmas de Gran Canaria (Spain) during the 2024/2025 academic year, including 325 students—204 from nursing and 121 from medicine—who completed a 7-item Likert-scale questionnaire assessing attitudes, perceived knowledge, and beliefs about the therapeutic potential, risks, and regulation of psychedelics, alongside sociodemographic data. Results revealed a growing interest in the therapeutic potential of psychedelics, although concerns persist. Gender and age were significantly associated with beliefs, with women reporting lower levels of perceived knowledge about psychedelics and older students exhibiting greater openness toward their therapeutic potential. Medical students demonstrated higher levels of perceived knowledge and greater agreement with therapeutic applications compared to nursing students, who more strongly associated psychedelic use with psychiatric risk. Participants with prior psychedelic use were more supportive of legalization and therapeutic use, highlighting the impact of personal experience. Formal education on psychedelics was linked to more favourable attitudes and increased knowledge, suggesting that training may reduce stigma and support evidence-based policy. Overall, students showed a cautiously optimistic view toward psychedelic therapies. These findings underscore the importance of integrating content on emerging treatments into health sciences curricula to foster informed, critical perspectives among future clinicians.

## Introduction

The World Health Organization (WHO) defines mental health as “a state of well-being that allows one to cope with the normal stresses of life, work productively and contribute to the community” [1]. Nowadays, mental health disorders represent a major public health challenge worldwide and constitute a leading cause of disability among young adults and university students [2]. In recent years, the burden of conditions such as anxiety and depression has increased substantially, a trend further intensified by the COVID-19 pandemic [3]. According to recent data, the prevalence of mental health problems in Spain has increased markedly in recent years. As a consequence, 42% of the Spanish population has used benzodiazepines in the past five years, with particularly concerning consumption rates among young adults aged 25–29 [4]. Current treatment strategies for mental disorders rely predominantly on pharmacological and psychotherapeutic interventions [5]. While these approaches are effective for many patients, their limitations—including partial treatment response, adverse effects, and concerns related to long-term use [6–9]—have prompted growing interest in alternative or adjunctive therapeutic strategies [10,11]. Within this context, psychedelic-assisted therapy have re-emerged as topics of scientific and clinical debate, raising important questions about their potential role in future mental healthcare and how they are perceived by upcoming generations of health professionals [12–14].

Psychedelic-assisted therapy involves the controlled administration of psychedelic compounds—typically in combination with psychotherapy—to facilitate emotionally meaningful and introspective experiences that may support the treatment of various psychiatric and psychological conditions, particularly anxiety and depression, for which growing evidence suggests potential efficacy and safety [15–17]. Classic psychedelics, such as psilocybin or lysergic acid diethylamide (LSD), exert their effects primarily through serotonergic pathways, particularly via agonism to 5-HT2A receptor [18]. In contrast, non-classical psychedelics such as 3,4-methylenedioxymethamphetamine (MDMA) act through different neurochemical mechanisms, including increased synaptic release of serotonin, dopamine, and noradrenaline, as well as modulation of oxytocinergic signalling [19–21].

Despite encouraging results, these therapies remain under regulatory evaluation in many countries. Emerging evidence suggests that public and professional attitudes toward psychedelic-assisted therapies may be shaped not only by clinical findings but also by broader drug policy frameworks and prevailing policy narratives. In this context, formal training programs have been developed for healthcare professionals, addressing past shortcomings such as overly punitive drug policies, and providing concrete education on the pharmacology, therapeutic potential, and risks of psychedelics, alongside practical guidance for safe and ethical implementation within clinical and research settings [22,23]. Shifts toward health-oriented policy approaches—such as prioritizing treatment and harm reduction over punitive measures—have been proposed as a factor that could influence public support for psychedelic reform [24,25]. Nevertheless, the therapeutic use of psychedelics is gradually gaining legal and institutional recognition in a growing number of jurisdictions. Several countries and regions have introduced regulatory frameworks that allow controlled clinical use, supervised therapeutic programs, or expanded research access, while psychedelic compounds continue to be investigated in clinical trials worldwide. In parallel, even in settings where these substances remain illegal, harm-reduction and integration-oriented practices have emerged to support individuals who use psychedelics outside formal medical contexts [10,26]. Regulatory constraints also extend to other health-related domains, including veterinary medicine, where emerging studies are beginning to explore the potential applications of psychedelic compounds in promoting animal welfare [27–29]. As regulatory, clinical, and research landscapes evolve, future healthcare professionals are increasingly likely to encounter psychedelic-assisted therapies, whether through direct clinical involvement, patient counselling, or interdisciplinary collaboration. This emerging context presents both opportunities and challenges, particularly considering the longstanding stigma associated with psychedelics and their historical classification as controlled substances. Understanding how future health professionals perceive these therapies is therefore essential to anticipate potential barriers and facilitators to their responsible integration into healthcare systems.

In this evolving context, medical and nursing students represent two distinct yet complementary groups of future healthcare professionals whose attitudes toward psychedelic-assisted therapies may shape the trajectory of their clinical integration and acceptance. In this context, perceptions of safety are particularly relevant, as public and professional concerns often derive from recreational use scenarios rather than from controlled clinical settings [30]. While classic psychedelics have demonstrated a favourable physiological safety profile and low toxicity when administered under medical supervision [10,17], potential risks—such as acute anxiety reactions, transient psychological distress, or belief and meaning alterations—remain central to ethical and clinical discussions [31,32]. Prior studies have focused on medical students [12], leaving a gap regarding nursing students’ perspectives. As psychedelic-assisted therapies continue to emerge within clinical and research settings, an increasing number of healthcare professionals—including physicians, nurses, psychologists, psychiatrists, and other allied health practitioners—are expected to be involved in their implementation.

This study aims to address this gap by comparatively examining the attitudes, beliefs, and factors associated with these perceptions among both medical and nursing students regarding psychedelic-assisted therapy. Understanding these differences and similarities is crucial for tailoring educational strategies and anticipating potential barriers or facilitators to the responsible adoption of these treatments across healthcare disciplines.

## Materials and methods

### Instrument

To assess the attitudes of nursing and medical students towards psychedelics, a previously published survey developed in English was used [12]. Although this instrument has not undergone formal psychometric validation in either English or Spanish, it has been employed in related studies assessing attitudes towards psychedelics [12,33,34]. For this study, the survey items were translated into Spanish using a forward and backward translation process to ensure conceptual and linguistic equivalence. The translation was performed by one of the co-authors and reviewed by the research team. Generative AI (ChatGPT, OpenAI, GPT-4.1-mini model) was used solely to assist with the correction of scientific English language and grammar during manuscript preparation. The authors remain fully responsible for the content and interpretation of the work. The Spanish-translated version of the survey, derived from the original English items, is provided in Appendix A for reference and to facilitate future research. The questionnaire consisted of seven items rated on a 5-point Likert scale (1 = strongly disagree; 2 = somewhat disagree; 3 = neither agree nor disagree; 4 = somewhat agree; 5 = strongly agree). The items assessed self-perceived knowledge about psychedelics, perceived psychiatric and cognitive risks, attitudes toward recreational and medical use, and support for further research, as follows:: 1) I would say I am knowledgeable about psychedelics; 2) The use of psychedelics increases the risk for subsequent psychiatric disorders; 3) The use of psychedelics should be illegal for recreational purposes; 4) The use of psychedelics is unsafe even under medical supervision; 5) The use of psychedelics shows promise in the treatment of psychiatric disorders; 6) The use of psychedelics may improve outcomes if used adjunctively with psychotherapy; and 7) The use of psychedelics deserves further research as potential treatment for psychiatric disorders.

In addition to attitudinal items, the survey collected sociodemographic and educational information, including age, gender, academic year, prior personal experience with psychedelics (specifically including MDMA, psilocybin, or LSD, which are among the most studied and recognized substances in current therapeutic research), and prior academic training on psychedelics. Regarding formal education, structured curricular content on psychedelics is limited and incorporated within the medical degree program at the University of Las Palmas de Gran Canaria, mainly through approximately one hour in the Toxicology course and three hours in the Drug Dependence course. No formal instruction on psychedelics is included in the nursing curriculum. However, students from both programs may voluntarily enrol in an interdisciplinary elective course on psychedelic therapies offered by the institution. We acknowledge that additional knowledge or training might have been obtained outside the formal curriculum through personal interests or extracurricular activities, which may have influenced participants’ self-perceived knowledge and attitudes. Attendance at any form of academic training on psychedelics was self-reported by participants.

The final version of the questionnaire was developed using the Google Surveys platform. To ensure anonymity, the collection of IP addresses, cookies, and any other information that could reveal the identity of participants was disabled. The survey link was distributed exclusively through the official communication channels of the Faculty of Health Sciences, sent to the institutional email accounts of enrolled medical and nursing students. This approach was intended to limit participation to the target population; however, given the nature of digital communication, absolute control over link sharing cannot be guaranteed.

The questionnaire included an informative paragraph explaining the study’s objectives, anonymity, and confidentiality of the data. Participation was entirely voluntary, with no form of compensation or negative consequences tied to either participation or refusal. Informed consent was obtained implicitly by participants actively confirming their agreement to participate before accessing the questionnaire. Due to the anonymous online nature of the study, the Ethics Committee waived the requirement for signed informed consent. Participants were also informed that they could discontinue their participation at any time without the need to provide a reason.

The data collection period extended from November 18, 2024, to January 20, 2025.

### Study population

Participants included all students officially enrolled in the Medicine and Nursing degree programs at the University of Las Palmas de Gran Canaria (Canary Islands, Spain) during the 2024/2025 academic year. There were no exclusion criteria. During the 2024/2025 academic year, official data provided by the Dean’s Office of the Faculty of Health Sciences indicated that 782 students were enrolled in the Medicine degree program and 547 in the Nursing degree program. Enrolment by academic year in Medicine was as follows: 118 (first year), 158 (second year), 136 (third year), 96 (fourth year), 147 (fifth year), and 127 (sixth year). In Nursing, the distribution was 104 (first year), 158 (second year), 143 (third year), and 142 (fourth year). All students were invited to participate in the study via official electronic communication from the Dean’s Office and through the dissemination of the survey link on social media platforms.

The study was approved by the Research Ethics Committee of the province of Las Palmas, Spain (ethical approval code #2024-446-1). All research procedures adhered to established ethical standards for studies involving human participants, in alignment with the principles set forth in the Declaration of Helsinki.

### Data collection and statistical analysis

To ensure data completeness, only fully completed questionnaires were accepted and analysed. The data were stored in Microsoft Excel (Microsoft Corporation, Redmond, WA, USA) and managed exclusively by the authors of the study. All information was used solely for research purposes, with no attempts made to identify or link responses to individual participants.

Likert-scale survey responses were treated as categorical variables for statistical analysis to avoid assuming equal intervals between response categories. This approach, which has been employed previously [28,29,35], allows for the examination of the distribution of responses across all categories without imposing parametric assumptions. Descriptive analyses of all variables were performed. For normally distributed continuous variables, means and standard deviations were reported, whereas medians and ranges were used for variables with non-normal distributions. The normality of the data was checked using the Kolmogorov–Smirnov test. Proportions were calculated for categorical variables. Since no continuous variable followed a normal distribution, comparisons between groups —specifically between medical and nursing students—were made using nonparametric tests (Mann–Whitney U test). Differences in categorical variables were analysed using the Pearson’s chi-square test. Since some response categories had sparse data, responses were consolidated into three broader categories (“Strongly disagree/Disagree,” “Neither agree nor disagree,” and “Agree/Strongly agree”) to ensure sufficient cell counts and facilitate interpretability. Additional analysis using the full five response categories was also performed, and corresponding results are provided for transparency. Degrees of freedom (df) for chi-square tests correspond to the number of response categories minus one. For analyses using the original 5-point Likert scale, df = 4; for analyses using the consolidated 3-category scale, df = 2. An alpha level of 0.05 was set as the threshold for statistical significance in all analyses. For database management and statistical analysis, the PASW Statistics software (version 19.0, SPSS Inc., Chicago, IL, USA) was used.

## Results

A total of 325 students completed the survey, including 121 medical students and 204 nursing students. Medical students represented 37.2% of the total sample, while nursing students accounted for 62.8%. Based on official 2024–2025 enrolment data, these responses correspond to a participation rate of 15.5% among medical students and 37.3% among nursing students. The median age was 21 years (range: 17–54), with no significant differences between medical and nursing students. Most participants were female (n = 258, 80.1%), with a significantly higher proportion in the nursing group (P < 0.001; Table 1). Fourth-year students were the most represented across both degrees, with 38.8% participation among those enrolled in that year. Prior psychedelic use was reported by 13 students (4.0%), with no significant differences by degree. Overall, 120 students (36.9%) reported having received formal academic training on psychedelics—significantly more among medical students (62.0%) compared to nursing students (22.1%) (P < 0.001; Table 1).

**Table 1:** Descriptive analysis of the whole series and segmented by study program (n, (%)).

	Whole series (n = 325)		Nursing (n = 204)	Medicine (n = 121)	P value*
Age (years)					0.635^#^
Median (range)^1^	21 (17–54)		21 (17–54)	21 (17–35)	
Gender					<0.001
Female	258 (79.4)		174 (85.3)	84 (69.4)	
Male	64 (19.7)		27 (13.2)	37 (30.6)	
Other	3 (0.9)		3 (1.5)	0 (0.0)	
Academic degree					NA
Nursing	204 (62.8)		204 (100)	0 (0.0)	
Medicine	121 (37.2)		0 (0.0)	121 (100)	
Academic year					0.187^‡^
1^st^	59 (18.2)		40 (19.6)	19 (15.7)	
2^nd^	67 (20.6)		48 (23.5)	19 (15.7)	
3^rd^	59 (18.2)		42 (20.6)	17 (14.0)	
4^th^	90 (27.7)		74 (36.3)	16 (13.2)	
5^th^	38 (11.7)		0 (0.0)	38 (31.4)	
6^th^	12 (3.7)		0 (0.0)	12 (9.9)	
Previous experience with psychedelics^2^					0.385
No	312 (96.0)		194 (95.1)	118 (97.5)	
Yes	13 (4.0)		10 (4.9)	3 (2.5)	
Academic training on psychedelics					<0.001
No	205 (63.1)		159 (77.9)	46 (38.0)	
Yes	120 (36.9)		45 (22.1)	75 (62.0)	
*Pearson’s chi-square test. Comparison between nursing and medical student groups. #Mann-Whitney U test. Comparison between nursing and medical student groups. ^‡^Only first- to fourth-year students were considered, as the nursing degree does not have a fifth or sixth year. ^1^The variable did not follow a normal distribution (Kolmogorov-Smirnov test; P value < 0.001) ^2^Referred to MDMA, psilocybin or LSD.					

The survey comprised seven items aimed at assessing students’ self-perceived knowledge and beliefs regarding psychedelics. In the whole series, more than half of the respondents (52.6%) reported limited self-perceived knowledge, as indicated by their disagreement or strong disagreement with the statement “I believe I am well informed about psychedelics” (Q1). In contrast, a large majority expressed concern about potential risks, with 76.3% agreeing or strongly agreeing that the use of psychedelics increases the risk of subsequent psychiatric disorders (Q2), and 72.0% endorsing the belief that recreational psychedelic use should remain illegal (Q3). Attitudes toward the safety and potential therapeutic use of psychedelics were more heterogeneous. A substantial proportion of respondents adopted a neutral position regarding the safety of psychedelics even under medical supervision (Q4; 40.3%) and their potential to improve outcomes when used adjunctively with psychotherapy (Q6; 45.2%). Notably, there was strong overall support for further scientific research, with 78.1% of participants agreeing or strongly agreeing that psychedelics deserve further investigation as potential treatments for psychiatric disorders (Q7).

These overall patterns were subsequently examined through stratified analyses by study program, sex, and age. In addition, the potential influence of formal academic training related to psychedelics and prior personal experience with these substances was explored.

### Influence of sex and age

**Fig 1:**
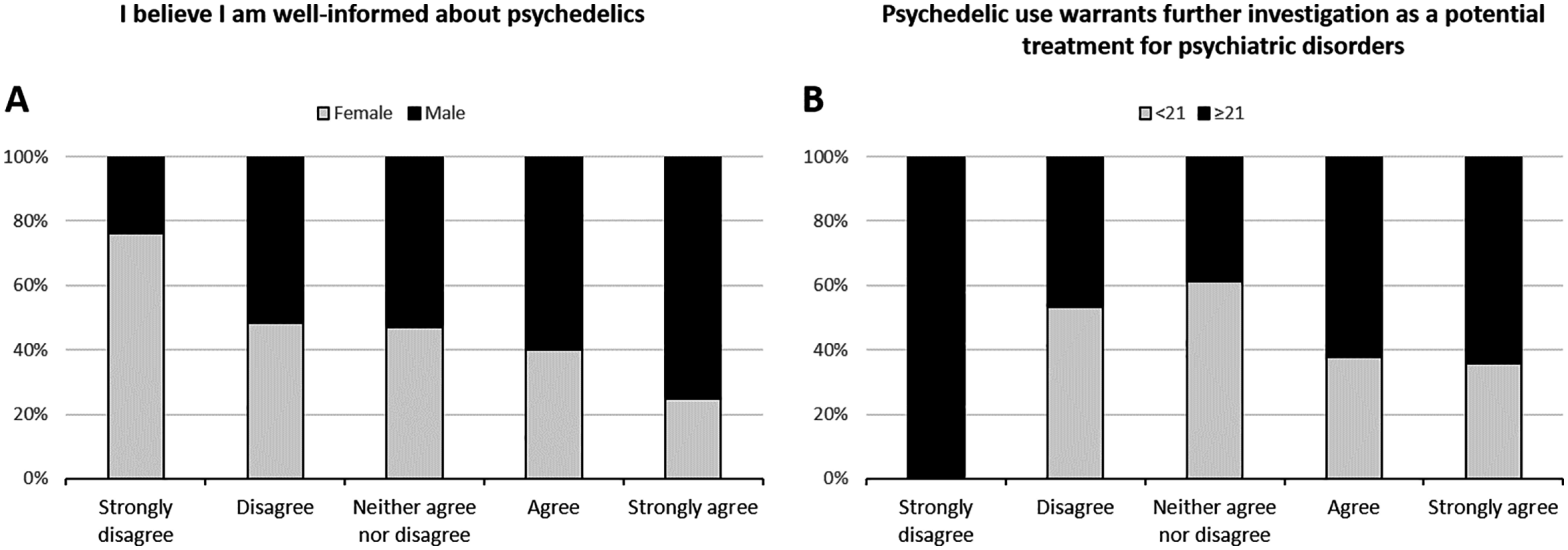
Stacked bar chart illustrating the distribution of responses to statements “I believe I am well-informed about psychedelics” (A) and “Psychedelic use warrants further investigation as a potential treatment for psychiatric disorders” (B), stratified by sex and age, respectively. Statistically significant differences were found in the distribution of responses: A, P value = 0.022; B, P value = 0.011 (Pearson’s chi square test).

Significant differences by sex were found only for Q1 (“I am well-informed about psychedelics”). A markedly higher proportion of women strongly disagreed with the statement compared to men (24.8% vs. 7.8%), whereas men were more likely to strongly agree than women (4.7% vs. 1.4%, respectively; P = 0.022; Fig 1A). Age showed a significant association with Q7 (“Psychedelic use warrants further investigation”). Among those under 21 years, 69.6% agreed or strongly agreed, compared to 84.2% of those 21 or older (P = 0.011; Fig 1B). Notably, the “strongly disagreed” category comprised only two responses overall. Both responses were provided by students in the older age group, whereas none of the younger participants selected this category. Consequently, within this specific response category, 100% of the responses corresponded to the older group. When treated as a continuous variable, younger students who agreed or strongly agreed had a median age of 19 years versus 22 in the older group (P = 0.026; Kruskal–Wallis test).

### Influence of study program

To facilitate interpretation and ensure sufficient cell counts for categorical analyses, responses to the seven survey statements were grouped into three categories: “Strongly disagree/Disagree,” “Neither agree nor disagree,” and “Agree/Strongly agree” (Table 2). For Q1 (“I am well-informed about psychedelics”), 31.4% of medical students (n = 38) agreed or strongly agreed, compared to just 14.2% of nursing students (n = 29; P < 0.001). Conversely, for Q2 (“Psychedelic use increases the risk of developing psychiatric disorders”), agreement was higher among nursing students (77.9%) than medical students (73.6%). In contrast, medical students showed significantly greater agreement with Q5 (“Psychedelics have shown promising results in treating psychiatric disorders”), Q6 (“Psychedelic use may enhance outcomes when combined with psychotherapy”), and Q7 (“Further research into therapeutic use is warranted”), with P values of 0.007, 0.006, and 0.047, respectively. It is important to note that the chi-square tests identify overall differences in response distributions between groups but do not specify which particular response categories drive these differences. No post hoc analyses were conducted to determine category-specific effects, and the results should be interpreted with caution.

**Table 2:** Analysis of the frequency distribution of survey responses stratified by study program.

Item	Nursing (n = 204)			Medicine (n = 121)			P value*
	Strongly disagree/disagree	Neither agree nor disagree	Agree/Strongly agree	Stronglydisagree/disagree	Neither agree nor disagree	Agree/ Strongly agree	
I1	130 (63.7)	45 (22.1)	29 (14.2)	41 (33.9)	42 (34.7)	38 (31.4)	<0.001
I2	5 (2.5)	40 (19.6)	159 (77.9)	11 (9.1)	21 (17.4)	89 (73.6)	0.027
I3	20 (9.8)	39 (19.1)	145 (71.1)	11 (9.1)	21 (17.4)	89 (73.6)	0.890
I4	76 (37.3)	83 (40.7)	45 (22.1)	52 (43.0)	48 (36.7)	21 (17.4)	0.478
I5	14 (6.9)	146 (71.6)	44 (21.4)	9 (7.4)	67 (55.4)	45 (37.2)	0.007
I6	18 (8.8)	105 (51.5)	81 (39.7)	9 (7.4)	42 (34.7)	70 (57.9)	0.006
I7	8 (3.9)	41 (20.1)	155 (76.0)	9 (7.4)	13 (10.7)	99 (81.8)	0.047

*Pearson’s chi square test.

I1: I believe I am well-informed about psychedelics.

I2: I believe that psychedelic use increases the risk of developing psychiatric disorders.

I3: I believe that recreational psychedelic use should remain illegal.

I4: The use of psychedelics is not safe, even under medical supervision.

I5: Psychedelic use has shown promising results in the treatment of psychiatric disorders.

I6: Psychedelic use may enhance treatment outcomes when used in conjunction with psychotherapy.

I7: Psychedelic use warrants further investigation as a potential treatment for psychiatric disorders.

To further assess whether collapsing response categories influenced the observed findings, an additional analysis using the full five-point Likert scale distribution was conducted and is presented in the S1 File. Overall, this analysis yielded results consistent with those obtained using the three-category grouping. Notably, the only exception concerned item Q2 (“I believe that psychedelic use increases the risk of developing psychiatric disorders”), for which the between-group difference did not retain statistical significance after disaggregation into five response categories.

Subgroup analyses explored the interaction between study program and sex (S2 File). Among nursing students, males were significantly more likely than females to report being well-informed about psychedelics (37.0% vs. 10.3%, P = 0.002). Comparing females across disciplines, medical students were more likely to report feeling informed (34.5% vs. 10.3%, P < 0.001), and nursing females more often agreed that psychedelics pose psychiatric risks (79.9% vs. 75.0%, P < 0.05). Medical females also more frequently endorsed the therapeutic value of psychedelics in Q5 and Q6 compared to nursing females (35.7% vs. 20.7% and 57.1% vs. 39.1%, respectively; P < 0.05). No significant differences were observed between male nursing and medical students. Age-related differences were also examined (S3 File). Among nursing students, those aged ≥21 years more often supported further research (Q7) than their younger peers (81.7% vs. 67.9%, P = 0.041). Medical students under 21 were more likely to feel informed (Q1: 29.4% vs. 10.7%, P < 0.01) and to perceive therapeutic promise (Q5: 35.3% vs. 17.9%, P < 0.05) than younger nursing students. These significant differences continued in older students, particularly for Q1 and Q6 (P < 0.001 and P < 0.01, respectively).

### Influence of formal academic training related with psychedelics

To assess the impact of formal education on perceptions of psychedelics, participants were divided into two groups: those with (n = 120) and without (n = 205) academic training on the topic (Table 3). Among those with formal education, 45.0% (n = 54) agreed or strongly agreed with the statement “I am well-informed about psychedelics” (Q1), compared to only 6.3% (n = 13) among those without training (P < 0.001). Similarly, agreement with “Psychedelic use has shown promising results in the treatment of psychiatric disorders” (Q5) was significantly higher among students with training (37.5% vs. 21.5%, P = 0.002), as was agreement with “Psychedelic use may enhance treatment outcomes when combined with psychotherapy” (Q6: 60.0% vs. 38.5%, P < 0.001). Of the 120 students with training, 111 evaluated its usefulness. Among them, 73.8% (n = 82) agreed or strongly agreed that the training was useful. No significant differences were found by sex, age, or study program. However, students who considered themselves well-informed (Q1) were significantly more likely to find the training useful (P = 0.001; S4 File). Specifically, 56.1% (n = 46) of those who felt well-informed also rated the training as useful, whereas only 9.7% (n = 8) showed a mismatch between these perceptions.

**Table 3:** Analysis of the frequency distribution of survey responses stratified by formal academic training related with psychedelics.

Item	Academic training, no (n = 205)			Academic training, yes (n = 120)			P value*
	Strongly disagree/ disagree	Neither agree nor disagree	Agree/ Strongly agree	Strongly disagree/ disagree	Neither agree nor disagree	Agree/ Strongly agree	
I1	151 (73.7)	41 (20.0)	13 (6.3)	20 (16.7)	46 (38.3)	54 (45.0)	<0.001
I2	7 (3.4)	39 (19.0)	159 (77.6)	9 (7.5)	22 (18.3)	89 (74.2)	0.259
I3	15 (7.3)	35 (17.1)	155 (75.6)	16 (13.3)	25 (20.8)	79 (65.8)	0.108
I4	75 (36.6)	88 (42.9)	42 (20.5)	53 (44.2)	43 (35.8)	24 (20.0)	0.357
I5	12 (5.9)	149 (72.7)	44 (21.5)	11 (9.2)	64 (53.3)	45 (37.5)	0.002
I6	19 (9.3)	107 (52.2)	79 (38.5)	8 (6.7)	40 (33.3)	72 (60.0)	<0.001
I7	11 (5.4)	39 (19.0)	155 (75.6)	6 (5.0)	15 (12.5)	99 (82.5)	0.298

*Pearson’s chi square test.

I1: I believe I am well-informed about psychedelics.

I2: I believe that psychedelic use increases the risk of developing psychiatric disorders.

I3: I believe that recreational psychedelic use should remain illegal.

I4: The use of psychedelics is not safe, even under medical supervision.

I5: Psychedelic use has shown promising results in the treatment of psychiatric disorders.

I6: Psychedelic use may enhance treatment outcomes when used in conjunction with psychotherapy.

I7: Psychedelic use warrants further investigation as a potential treatment for psychiatric disorders.

### Influence of previous experiences with psychedelics

**Fig 2:**
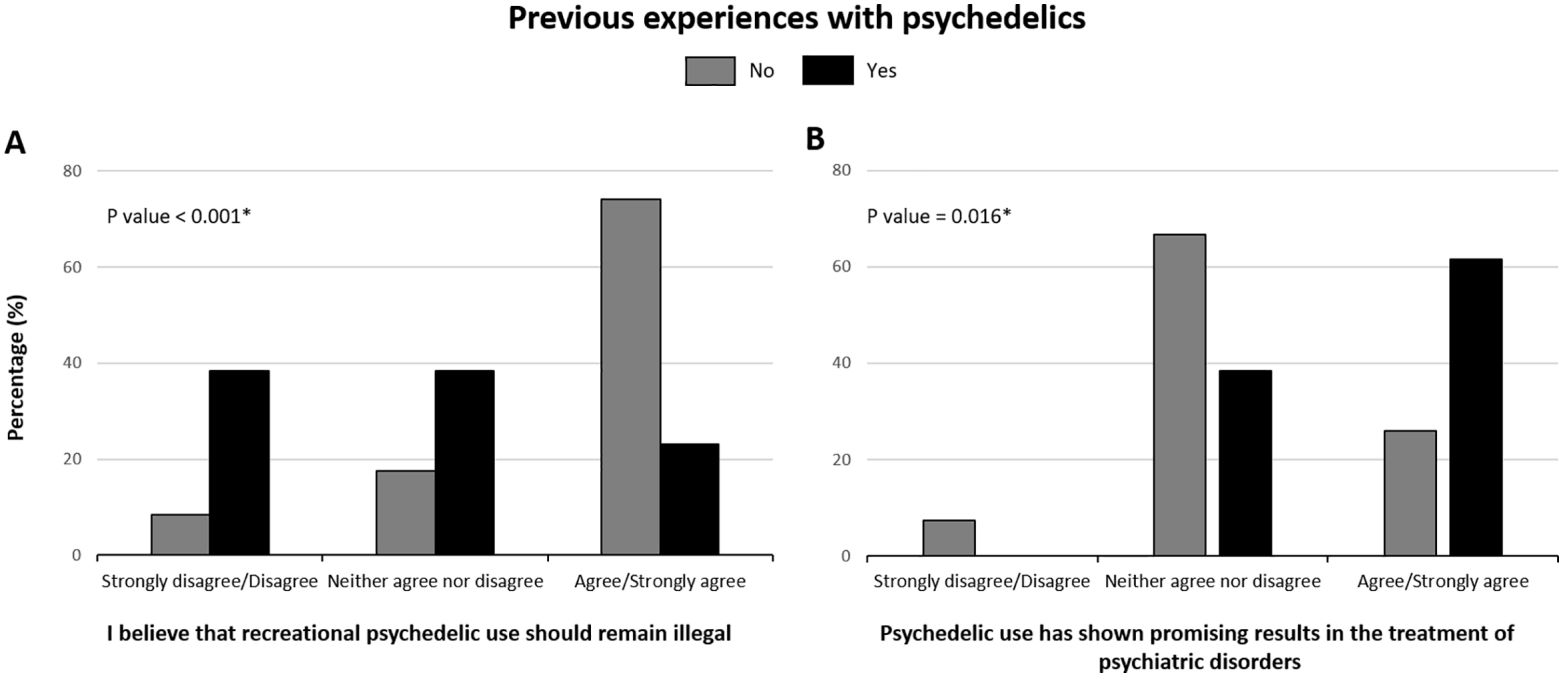
Bar chart illustrating the distribution of responses to statements “I believe that recreational psychedelic use should remain illegal” (A) and “Psychedelic use has shown promising results in the treatment of psychiatric disorders” (B), stratified by prior psychedelic use. *Pearson’s chi square test.

Finally, we explored how prior psychedelic experience influenced participants’ attitudes (Fig 2). Among the 13 students with such experience, 38.5% disagreed/strongly disagreed with the statement “Recreational psychedelic use should remain illegal” (Q3), compared to only 8.3% among those without experience (P < 0.001; Fig 2A). Additionally, 61.5% of experienced users agreed/strongly agreed with “Psychedelic use has shown promising results in treating psychiatric disorders” (Q5), versus 26.0% of non-users (P = 0.016; Fig 2B). Notably, none of the experienced participants disagreed with Q5, suggesting a more favorable perception of therapeutic potential among this subgroup.

## Discussion

This study analysed the attitudes and beliefs of medical and nursing students regarding the therapeutic use of psychedelics. Overall, participants showed limited knowledge about these substances. While there was optimism about their potential benefits, this was accompanied by caution due to perceived risks. Additionally, sociodemographic factors such as sex and academic background were significantly associated with these perceptions, reflecting differences in self-reported knowledge and attitudes toward psychedelics among subgroups.

In terms of general attitudes, most participants in the present study expressed support for continued research on psychedelic-assisted therapies, with 78.1% agreeing or strongly agreeing that psychedelics deserve further investigation (Q7). When analyses were restricted to medical students, this proportion increased to 81.8%, closely aligning with findings from the only previously published study specifically examining medical students’ attitudes toward psychedelics [12], conducted in the United States (state of Nevada), in which 95.2% of respondents (n = 132) endorsed further research in this field. Although support was slightly lower in our sample, this consistency across studies suggests a robust and growing acceptance of psychedelic research among future physicians. Similarly, attitudes regarding the therapeutic potential of psychedelics (Q5) showed comparable patterns. In the previous study, 78.6% of medical students agreed or strongly agreed that psychedelics could be useful in the treatment of psychiatric disorders [12], whereas in our medical student subsample, 73.5% expressed agreement with this statement. Despite differences in institutional context (e.g., university location and health system regulations) and sample characteristics (e.g., age distribution, proportion of female students, and prior exposure to formal training), these convergent findings reinforce the perception of psychedelics as promising therapeutic agents among medical trainees. In contrast, perceptions of psychiatric risk differed more markedly between studies. While only 35.0% of medical students in the previous study agreed that psychedelic use increases the risk of psychiatric disorders [12], this proportion was slightly higher in our medical student subsample (37.2%) and even more pronounced in the overall sample (76%). These discrepancies may reflect contextual factors such as differences in educational exposure, cultural attitudes, or the inclusion of nursing students in the present study, who tended to express greater concern regarding psychiatric risks. Importantly, this coexistence of optimism regarding therapeutic potential and concern about safety suggests a nuanced and critical stance rather than uncritical enthusiasm. Such a balanced perspective is consistent with the broader scientific literature, which highlights both the potential benefits of psychedelics and the need for careful screening, supervision, and regulatory oversight [31,36].

Regarding gender differences, women reported significantly lower levels of perceived knowledge about psychedelics. This disparity may be more plausibly explained by differences in prior exposure to psychedelic-related information or training—whether academic or experiential—rather than by general differences in self-assessed competence, although broader social and educational factors cannot be entirely excluded. Such an interpretation is consistent with previous research indicating that perceived knowledge in this field is strongly associated with direct educational or experiential contact [37]. Additionally, the higher prevalence of prior psychedelic use among men may contribute to increased exposure to the topic, thereby enhancing their perceived knowledge. In general, the prevalence of drug use is higher among men, which may influence their attitudes toward these substances [38]. However, given that the data reflect self-reported perceptions, it is not possible to conclude whether actual knowledge differs between groups. Future research should consider incorporating more explicit objective measures to assess factual knowledge about psychedelics. While several items in the present questionnaire (e.g., those addressing psychiatric risk, safety under medical supervision, and therapeutic efficacy) are directly informed by existing scientific evidence, and thus indirectly reflect participants’ alignment with the current literature, dedicated knowledge-based assessments could allow for a clearer distinction between attitudes, misconceptions, and evidence-informed understanding, particularly in areas where high-quality clinical data are already available (e.g., randomized controlled trials demonstrating the efficacy of psilocybin-assisted therapy in treatment-resistant depression [39]).

In the present study, medical students reported higher perceived knowledge compared to nursing students. They also showed greater agreement with the therapeutic potential of psychedelics, while nursing students expressed more concern about associated risks. Another important factor shaping students’ attitudes is that 36.9% of the sample had received formal training on psychedelics. Those with training were significantly more likely to recognize the therapeutic benefits of psychedelics combined with psychotherapy. Thus, the observed differences between medical and nursing students in perceived knowledge and endorsement of the therapeutic potential of psychedelics should be interpreted with caution. It should be emphasized that, although formal education on psychedelics is officially included only in the medical curriculum, a non-negligible proportion of nursing students (22.1%) reported having received some form of training on this topic. In contrast, this percentage was substantially higher among medical students (62.0%). This finding suggests that exposure to psychedelic-related education is not exclusively determined by official curricula but may also occur through extracurricular courses, seminars, or self-directed learning. It is important to consider that the nursing cohort included a substantially higher proportion of female students, who concurrently reported lower levels of training on psychedelics compared to their medical counterparts. This overlap between gender distribution and educational exposure may act as confounding factors, complicating the attribution of differences solely to gender or solely to previous formal training on psychedelics. Nevertheless, the marked difference between both groups supports the interpretation that formal curricular inclusion plays a significant role in shaping knowledge and attitudes toward psychedelics. To our knowledge, this is the first study comparing students from different health-related programs within the same academic institution in terms of attitudes and knowledge about psychedelic therapy. This within-institution comparison allows for a more controlled assessment of program-specific educational and cultural influences, minimizing variability related to institutional context. Considering nurses’ critical role in psychedelic-assisted therapy and in offering emotional support throughout patient recovery, proper training is vital to alleviate unwarranted concerns and foster evidence-based clinical practice.

Personal experience with psychedelics plays a significant role in shaping attitudes toward their use. Prior exposure tends to correlate with more favourable views regarding the therapeutic potential of these substances and a reduced support for the prohibition of recreational use. This interpretation is consistent with previous research conducted in Spanish and Spanish-speaking populations, which highlights the role of direct experience and contextual factors in shaping perceptions of psychedelic substances beyond purely recreational frameworks [30,32]. In a large cross-sectional study of the general population, motivations for psychedelic use (set), along with contextual variables such as natural settings and the presence of trusted companions (setting), were associated with lower levels of psychopathology, greater wellbeing, and more meaningful experiences, whereas problematic or recreational motivations predicted poorer psychological outcomes [30]. These findings support the notion that perceptions of psychedelics are strongly influenced by the context in which they are understood and discussed, particularly when therapeutic and recreational uses are clearly differentiated. Similarly, a second study examining patterns of psychedelic use across Spanish and South American populations identified significant influences of age and sex on consumption patterns and adverse effects, with younger age and male sex associated with higher risk profiles and predominantly recreational use [32]. Although these studies focused on users rather than healthcare students, their results align with our findings by underscoring how exposure, contextual framing, and demographic variables may shape attitudes toward psychedelics and their perceived therapeutic potential. Such evidence suggests that first-hand experience may contribute to more positive attitudes toward the development and acceptance of psychedelic-based therapies. However, it is also important to acknowledge the potential limitations associated with first-hand experience. Prior work has cautioned that direct exposure to psychedelics may foster positive expectancy effects and cognitive biases, including overly favourable attitudes toward legalization and the underestimation or neglect of potential psychological, ethical, and clinical risks. Conceptual and ethical analyses have highlighted concerns regarding the influence of personal psychedelic experiences on professional judgment, including the risk of introducing spiritual, ideological, or experiential biases into clinical practice and research interpretations [40]. In this context, the assumption that first-person experience necessarily enhances epistemic or therapeutic competence has been questioned, particularly in the absence of robust empirical evidence supporting its unique educational value [41]. These considerations underscore the need to balance experiential familiarity with rigorous scientific training, ethical safeguards, and critical appraisal, especially when evaluating attitudes toward the therapeutic use of psychedelics among future healthcare professionals.

### Strengths and limitations

This study presents several limitations. First, the sample was restricted to students from a single institution, which limits the generalizability of the findings to other academic and cultural contexts. Although psychologists play a central role in psychedelic-assisted therapies, this population could not be included in the present study because a Psychology degree is not offered at our institution. Future multicenter studies including psychology students and other health professionals would provide a more comprehensive view of interdisciplinary attitudes toward psychedelic therapy. Second, the use of self-reported measures may be subject to social desirability bias, and the voluntary nature of participation may have resulted in the overrepresentation of students with a particular interest in psychedelics. Third, although the survey was distributed via official institutional channels targeting enrolled medical and nursing students, the online nature of the dissemination means that we cannot fully exclude the possibility that the survey link was forwarded outside the intended population. This potential lack of control over the sampling frame represents a limitation to the study’s internal validity. Fourth, although Likert-scale items were appropriately analyzed as categorical variables using chi-square tests to detect overall differences in response distributions between groups, this approach does not allow for the identification of specific response categories driving those differences without additional post hoc analyses. Consequently, the results should be interpreted as indicating the presence of distributional differences between groups rather than category-specific or directional effects. Additionally, no corrections for multiple comparisons were applied in the analyses, which may increase the risk of type I error. This limitation should be considered when interpreting the findings. Notably, the reference study that informed our questionnaire design also did not perform multiple comparison corrections, providing some context for this methodological choice [12]. An additional limitation concerns differential exposure to formal education on psychedelics between academic programs. Medical students receive limited curricular content on this topic (approximately one hour within a Toxicology course and three hours within a Substance Use Disorders course), whereas nursing students do not receive formal instruction within their degree program. Although this exposure is quantitatively modest, it may have influenced perceived knowledge and attitudes and should be considered when interpreting group differences. Importantly, this disparity reflects current educational structures rather than an experimental manipulation. Future research should include students from multiple universities and disciplines to enhance external validity, employ longitudinal designs to examine how attitudes evolve across academic training, and incorporate objective measures of psychedelic-related knowledge to complement self-reported assessments.

Despite these limitations, the study has several strengths. The topic addressed is both novel and of increasing scientific and societal relevance. A survey instrument previously employed in related studies was used, providing a sound methodological foundation. Including both nursing and medical students allowed for meaningful comparisons across disciplines, thereby enhancing the depth of analysis. Additionally, the integration of demographic, educational, and experiential variables allowed for a nuanced understanding of factors associated with students’ perceptions and attitudes. These analyses revealed significant differences that strengthen the relevance and implications of the findings.

## Conclusions

This study reveals an interest among Health Sciences students in the therapeutic potential of psychedelics, alongside persistent concerns about their safety. The findings underscore the crucial role of structured academic education in shaping informed and critical attitudes toward these substances. Notably, medical students—among whom a higher proportion reported receiving formal education on psychedelics—tended to demonstrate greater awareness and acceptance, whereas nursing students, with less reported formal education, expressed comparatively more caution. Given the key role of nurses in the administration and emotional support of emerging psychedelic therapies, integrating targeted training into nursing curricula is essential for their safe and effective implementation.

## Supporting information

S1 Appendix Spanish-translated survey items. (DOCX)

S1 File Analysis of the frequency distribution of survey responses (n, (%)) by study program. (XLSX)

S2 File Frequency distribution of survey responses (n, (%)), stratified by study program and sex, with comparisons performed within each study program between sexes. (XLSX)

S3 File Frequency distribution of survey responses (n, (%)), stratified by study program and age group (below vs. above the median age), with comparisons performed within each study program across age groups. (XLSX)

S4 File Bar chart showing the distribution of responses regarding the perceived usefulness of formal academic training on psychedelics in relation to their self-reported knowledge about these substances. The x-axis represents the perceived knowledge about psychedelics, while the y-axis represents the perceived usefulness of the training. To highlight the association between the two variables, a line was used to represent individuals who agreed/strongly agreed with the usefulness of the training. A secondary y-axis has been added to the right of the graph to represent the absolute values of these data set.
(TIF)
