# Supplementary material for: Attitudes toward psychedelic therapy among medical and nursing students: A cross-sectional survey study
Source: PLoS One. 2026 Mar 31;21(3):e0344698. doi: 10.1371/journal.pone.0344698 (PMC13037965; doi:10.1371/journal.pone.0344698)
Supplement: S1 Appendix — (DOCX) [file pone.0344698.s001.docx]

| **Item number** | **English** | **Spanish** |
| --- | --- | --- |
| **1** | I would say I am knowledgeable about psychedelics. | Diría que estoy bien informado sobre psicodélicos. |
| **2** | The use of psychedelics increases the risk for subsequent psychiatric disorders. | Creo que el uso de psicodélicos aumenta el riesgo de padecer trastornos psiquiátricos. |
| **3** | The use of psychedelics should be illegal for recreational purposes. | El uso de psicodélicos debería ser ilegal para fines recreativos. |
| **4** | The use of psychedelics is unsafe even under medical supervision. | El uso de psicodélicos no es seguro ni siquiera bajo supervisión médica. |
| **5** | The use of psychedelics shows promise in the treatment of psychiatric disorders. | El uso de psicodélicos muestra resultados prometedores en el tratamiento de trastornos psiquiátricos. |
| **6** | The use of psychedelics may improve outcomes if used adjunctively with psychotherapy. | El uso de psicodélicos puede mejorar los resultados si se utilizan de forma complementaria a la psicoterapia. |
| **7** | The use of psychedelics deserves further research as potential treatment for psychiatric disorders. | El uso de psicodélicos merece más investigación como posible tratamiento para los trastornos psiquiátrico. |

**Appendix A. Spanish-translated survey ítems.**
