# Supplementary figures and images for: Attitudes toward psychedelic therapy among medical and nursing students: A cross-sectional survey study
Source: PLoS One. 2026 Mar 31;21(3):e0344698. doi: 10.1371/journal.pone.0344698 (PMC13037965; doi:10.1371/journal.pone.0344698)

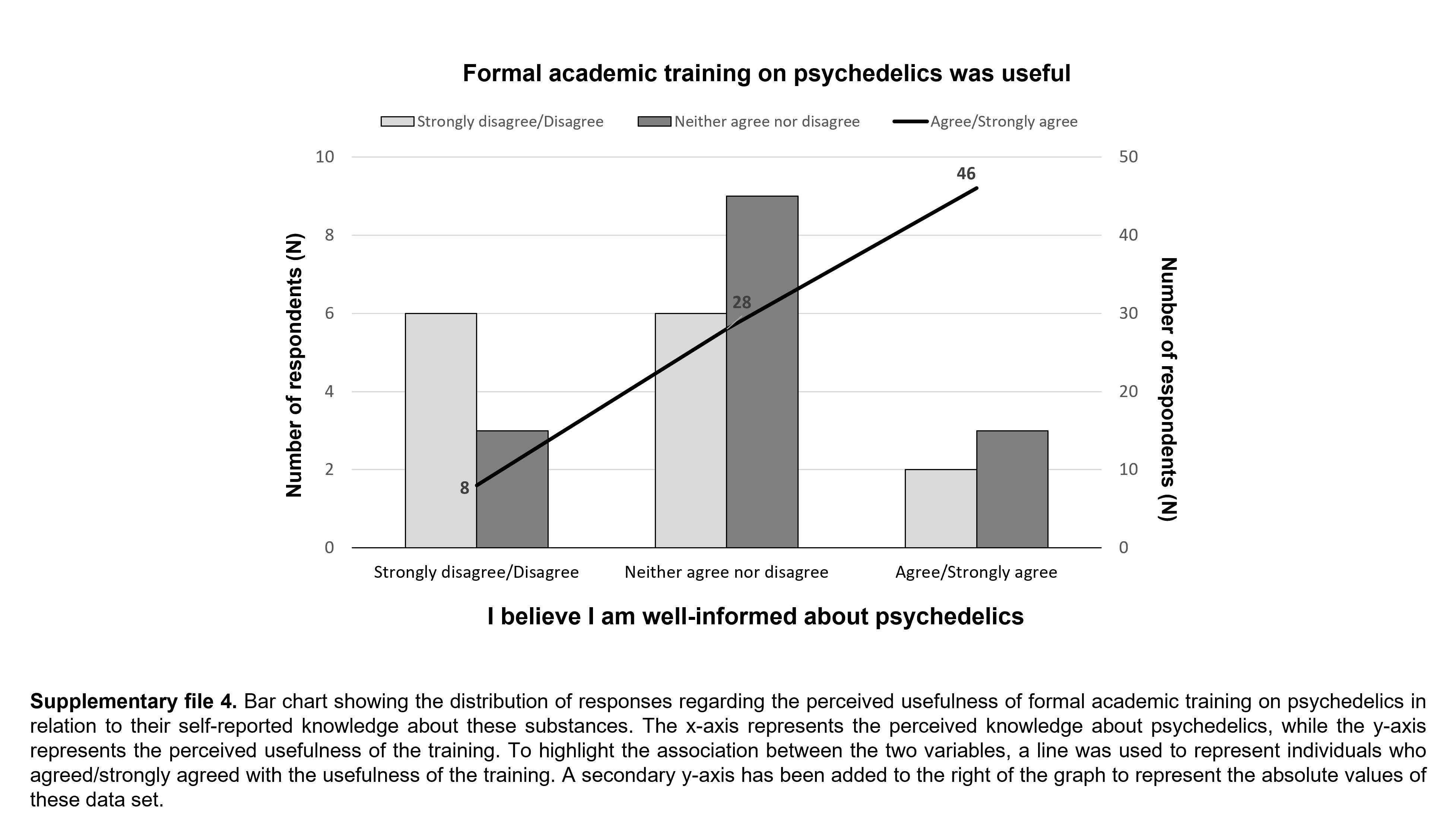

Supplement: S4 File — The x-axis represents the perceived knowledge about psychedelics, while the y-axis represents the perceived usefulness of the training. To highlight the association between the two variables, a line was used to represent individuals who agreed/strongly agreed with the usefulness of the training. A secondary y-axis has been added to the right of the graph to represent the absolute values of these data set. (TIF) [file pone.0344698.s005.tif]
